# Direct Anterior Approach: The Outlook of Total Hip Arthroplasty in Crowe Type III–IV Hip Dysplasia

**DOI:** 10.1111/os.12672

**Published:** 2020-05-05

**Authors:** Yuan Zhang, Xia Zhang, Yue Zhou

**Affiliations:** ^1^ Joint Disease & Sport Medicine Center, Department of Orthopedics, Xinqiao Hospital Army Medical University Chongqing China

Dear Editor,

We write this letter in response to the article titled “Total Hip Arthroplasty for Crowe Type IV Hip Dysplasia: Surgical Techniques and Postoperative Complications” by Shi *et al*.^1^ published in the latest issue of *Orthopaedic Surgery* (2019;11:966–973). This is a valuable review article focusing on the current concerns and controversies in treating Crowe type IV developmental dysplasia of the hip (DDH). Through distinctive hypotheses, comprehensive viewpoints, and a variety of documented evidences, the article's logic and views are presented clearly. First, the authors realized the inadequacy of traditional approaches and techniques in managing Crowe type IV DDH, and assumptively introduced the direct anterior approach (DAA) to total hip arthroplasty as a surgical strategy. They also underlined the theoretical advantage of DAA in Crowe type IV DDH, and further predicted how DAA could produce satisfactory treatment outcomes for this type of population. In particular, an extraordinary work by Oinuma *et al*.^2^ was referenced in the article to further validate the authors’ point of view. Unfortunately, the authors have not fully revealed the technical challenges in treating Crowe type IV DDH via DAA, or the deficiency of the surgical technique proposed by Oinuma *et al*. Further, we did not find any insights or advice for optimizing the technique or the procedure, nor did we find a clinical result from the authors' practice. That is the reason behind writing this Letter to the Editor, and we would like to systemically discuss these questions in the latter part of this letter.

Second, the authors introduced some current debates about traditional approaches to total hip arthroplasty: it is still highly controversial whether the rotation center should be reconstructed in the true acetabulum or placed at the dislocated position. Through their analysis, the authors conveyed an important message; that high hip center technique, as much as 30–35 mm in height, resulted in relatively satisfactory outcomes in Crowe type III DDH, as the studies referenced in the article showed. This finding sheds light on a precise compromise between anatomical reconstruction in the hip center and coverage‐induced stability of the acetabular component, which might be very crucial in the clinical decision‐making of Crowe type III to IV DDH. The authors then switched to the decision‐making process of femoral osteotomy scheme in Crow type IV DDH. Five types of femoral osteotomy techniques – including the transverse, the oblique, the step‐cut, the double chevron, and the sigmoid osteotomies – were illustrated in schematic diagrams and elaborately discussed in terms of their rationality, feasibility, and reproducibility; however, the value and advantage of greater trochanter osteotomy was neglected in the article. Actually, the greater trochanter osteotomy was widely employed as an effective method in the early stage of hip arthroplasty, which also contributed to the advancement of total hip arthroplasty to this modern attitude. We have to admit that both the classical sliding osteotomy^3^, and proximal femoral reconstruction technique proposed by Shang *et al*.^4^, provide efficient and reliable solutions for femoral reconstruction procedure in patients with Crowe type III‐IV DDH, as well as minimal risks of bone non‐union and dislocation.

Third, common complications including leg length discrepancy, intraoperative fracture, bone non‐healing, nerve palsy, and the pathological mechanism were generally described. In fact, the occurrence of these complications is considered to be closely related to the surgical approach. To this point, the authors should have allowed more space to propagate the benefit of direct anterior approach for high‐dislocated DDH.

As to our best knowledge, there are very few reports to describe the technical issues and summarize the outcomes in treating high‐dislocated DDH by DAA. The extreme challenges in this procedure might include multiple planar deformities in both acetabulum and proximal femur, the complex lumbar‐pelvis‐hip pathology, demanding reconstruction and correction techniques, and modular stem implant difficulties^5^, ^6^, ^7^. However, the study by Oinuma *et al*. firstly reported the early outcomes of nine patients diagnosed with Crowe type IV DDH by DAA, combined with subtrochanteric shortening osteotomy in Japan from 2006 to 2011^2^. Despite their impressive technique, the limitation of this study is the undesirable intraoperative variables and clinical outcomes compared to those of the posterolateral approach, which might be attributed to their aggressive manipulation in exposing the proximal femur, and extensive damage of the short external muscles, especially the obturator internus and the superior and inferior gemelli.

There is growing demand for DAA from both patients and surgeons to solve DDH pathologies in Southwest China due to high morbidity in this region. Although the exact etiology of DDH is not clear, some intrinsic factors have been suggested to contribute to its progression, including genetic (short and thin body shapes), cultural (swaddling, kneeling), geographic (mountainous areas), and climatic (less sunshine, hot and moist) factors^8^, as well as less‐developed economies and healthcare systems.

In this letter, we are glad to report that our pioneering practice of surgical techniques and standardized protocol has been established in our teaching hospital in treating high‐dislocated DDH by DAA. Up to 20 cases of Crowe type III‐IV DDH have been implemented via DAA in the past 3 years, with comparable intra‐ and postoperative complications as traditional approach. An improved clinical outcome was observed during a short‐term follow‐up (2 years), as evidenced by faster recovery of hip abductors and flexor muscle strength, shorter time to regain a normal gait, higher hip function scores in Harris, WOMAC, and SF‐12 scales, and more accurate component position, as compared to traditional posterolateral approach. We do believe that a better long‐term survival of the prosthesis could be anticipated by this surgical technique.

Our advancement in treating high‐dislocated DDH, as compared to Oinuma *et al*., is a modified surgical technique and standardized procedure, which includes individualized preoperative planning (such as algorithm of limb length equalization), specific soft tissue release techniques (such as peeling‐off of tensor fasciae latae, pie‐crusting of the iliotibial band, sleeve‐like whole capsulectomy), as well as an innovative subtrochanteric shortening, de‐rotation, or oblique osteotomy via direct anterior approach. Our technique could maximally maintain the integrity of dynamic stabilizer around the hip (especially the short external rotators), significantly reduce the surgical trauma, and further promote postoperative recovery. Additionally, some advanced technologies and equipment, such as intraoperative computed tomography (CT), computer navigation, and nerve monitoring, are introduced for some specific cases to guarantee intraoperative accuracy and safety. We would like to submit and publicize these essential techniques in the forms of a clinical research article, video in orthopaedic technique, and instructional course in the coming issues of *Orthopaedic Surgery*. We believe this series of work will be notably imperative to overcome the current difficulties in treating high‐dislocated DDH by DAA.

As Shi and his colleagues mentioned in their article, the learning curve and intraoperative complication were the key concerns to start treating high‐dislocated DDH via DAA. Our success is based on the evolution of the surgical technique formulated by treating over 300 cases of DDH. Our team have treated over 1200 cases of THA using the DAA since 2015, including over 300 cases of osteoarthritis secondary to DDH. Therefore, DAA is a good strategy for most surgeons to start performing in patients with Crowe type I‐II DDH, and then gradually in patients with Crowe type III or even type IV DDH.

In our philosophy, although most studies have shown satisfactory mid‐ to long‐term clinical results by traditional posterolateral‐approach THA in treating high‐dislocated DDH^9^, ^10^, some major deficiencies should not be ignored. First, invasion of the short externals, although properly repaired, might increase the risk of instability and dislocation^11^. Second, interruption of the branch of the femoral artery impairs osteointegration on the host bone‐prothesis interface and increases the nonunion at the osteotomy site and failure rate of femoral stem^12^. Because of the inter‐nerve and inter‐muscle pathway of DAA, the above muscles and vessels could be maintained intact, which further ensure the accelerated recovery of the hip function and osteointegration between bone and prosthesis.

Furthermore, there are several unproven but observed properties of DAA versus the traditional approach in treating high‐dislocated DDH. For example, the lumbar‐pelvic‐hip complex plays a key role in decision‐making for Crowe type III‐IV DDH 13. Sagittal imbalance of the pelvis usually causes anterior or posterior impingement during sit‐to‐stand transition^14^. Therefore, a functional pelvic position can be more effectively achieved in the supine position with the DAA than in the lateral decubitus position with the posterolateral approach. A functional pelvic position is considered crucial for reproducible and safe acetabular component sizing and positioning. Therefore, the superiority of the optimum component position, impingement‐free motion, and stability of the hip can be easily obtained with DAA, which has been proven in ample studies and our own practice.

Conclusively, the theoretical advantage in treating high‐dislocated DDH by DAA was underlined by Shi and his colleagues. In addition, our clinical practice suggested that the DAA exhibited positive properties of minimally invasive and fast‐recovery surgery, reduced interruption of the stabilizers around hip, provided more impingement‐free motion and stability of the hip, and allowed for optimal positioning of the components. Therefore, we are full of confidence that DAA will be the future of total hip arthroplasty for Crowe III‐IV DDH.

## Disclosure

None of the following authors or any immediate family member has received anything of value from or has stock or stock options held in a commercial company or institution related directly or indirectly to the subject of this article. All of the authors confirm that there is no conflict of interest.
